# Assessing the Interfertility of Sexual *Chara canescens* Populations from Europe: Difficulties and Challenges

**DOI:** 10.3390/plants15213434

**Published:** 2026-11-04

**Authors:** Adriana Arnal, María A. Rodrigo, Pablo García-Murillo, Karl-Georg Bernhardt, Riccardo Guarino, Dario Salemi, Angelo Troía, Barbara Turner, Johanna Weitzel, Hendrik Schubert

**Affiliations:** 1 Cavanilles Institute for Biodiversity and Evolutionary Biology (ICBiBE), University of Valencia, 46980 Paterna, Spain; maria.a.rodrigo@uv.es; 2 Department of Plant Biology and Ecology, Faculty of Pharmacy, University of Sevilla, 41012 Seville, Spain; pgarcia@us.es; 3 Department of Ecosystem Management, Climate and Biodiversity, BOKU-University of Natural Resources and Life Sciences, 1180 Vienna, Austria; karl-georg.bernhardt@boku.ac.at (K.-G.B.); barbara.turner@boku.ac.at (B.T.); 4 Department of Biological, Chemical and Pharmaceutical Sciences and Technologies (STeBiCeF), University of Palermo, 90128 Palermo, Italy; riccardo.guarino@unipa.it (R.G.); dario.salemi@unipa.it (D.S.); angelo.troia@unipa.it (A.T.); 5 Department of Earth and Marine Sciences (DiSTeM), University of Palermo, 90123 Palermo, Italy; 6 National Biodiversity Future Center (NBFC), Piazza Marina 61, 90133 Palermo, Italy; 7 Institute for Biosciences, Aquatic Ecology, University of Rostock, 18059 Rostock, Germany; johanna.weitzel@uni-rostock.de (J.W.); hendrik.schubert@uni-rostock.de (H.S.)

**Keywords:** charophytes, cross-fertilisation, parthenogenesis, F_0_ and F_1_ generations, reproductive isolation, germination success

## Abstract

Most known populations of the charophyte *Chara canescens* consist entirely of parthenogenetic reproducing females. Conversely, sexual populations are quite rare. A European project is trying to unravel the degree of genetic exchange between *C. canescens* populations. As part of this issue, interfertility experiments (cross-fertilisation) of females with males from different sites were planned. Of 72 potential combinations, seven were successfully performed, in five of them oospores were produced, and only supposedly cross-fertilised oospores appeared from one crossing (Sechsmahdlacke’s males, Austria x Pauli Murtas’ females, Italy). Among the main drawbacks faced (i) using specimens directly from the field are the low number of sexual populations in Europe; the long distances between sexual populations to obtain specimens simultaneously for the crossings; the low number of male individuals in the populations; and the difficulties in getting “healthy” cultures in the laboratory; whereas, (ii) by using specimens germinated in the laboratory: the relatively low number of germinated individuals in general; the very low number of germinated male individuals; the long time needed for the germination from the sediment oospores and the asynchronous appearance between populations of different origin; and, finally, the possible sympatric existence of both types of females in the same site, and the impossibility of distinguishing among them visually. Regarding this last issue, sympatric occurrence of *C. canescens* sexually and parthenogenetically reproducing females is confirmed for the first time in the Las Eras water body (Spain). This meant not knowing whether the oospores formed in the three crossings with females from this site were parthenogenetically or sexually produced. Although the interfertility results remain inconclusive regarding reproductive isolation among distant *C. canescens* populations, the methodological framework presented here provides a solid foundation for future research.

## 1. Introduction

*Chara canescens* Loiseleur 1810 is a complex green alga within the family Charophyceae, belonging to the division Streptophyta [1], and it is closely related to land plants. Globally, this charophyte species is widely distributed in the northern hemisphere. The main occurrences are in Europe, but *C. canescens* is also found in North America, Asia, and northern Africa. South of the equator, it has only been recorded in Australia [2,3].

*C. canescens* is the only charophyte species capable of reproducing parthenogenetically [2]. Moreover, it is unique in exhibiting two different reproductive strategies (parthenogenetic and sexual). Populations consisting exclusively of female individuals, and reproducing apomictically, account for a large part of the aforementioned wide distribution of *C. canescens* [2]. On the other hand, the sexual populations constitute only a very small proportion of the overall distribution (to date, only seven such occurrences have been confirmed; see [4]. This phenomenon was first described by Brown in 1856 [5] as ‘geographic sex separation’, later named ‘geographic parthenogenesis’ by Vandel in 1928 [6].

The parthenogenetic populations have the advantage of not being dependent on fertilisation and of being able to colonise new habitats rapidly [7]. However, this type of reproduction also carries the risk of genetic impoverishment, as there is no genetic recombination in the absence of meiosis and fertilisation. As a result, such populations have a lower potential for acclimatisation and may quickly enter an extinction vortex [8,9]. Nevertheless, Schaible et al. [10], in their analysis of the genetic diversity of parthenogenetic and sexual populations of *C. canescens*, revealed that parthenogenetic populations are more genetically diverse than would be expected based on their reproductive strategy. A hypothesis is that this is due to the introduction of oospores from sexually reproducing populations into parthenogenetic populations, suggesting some degree of genetic exchange. Among the ecological roles the charophytes perform in aquatic environments [11] is the provision of a valuable food supply for birds, by serving both as a habitat for prey species and as a food source themselves [12,13]. This is relevant for the charophyte population dynamics, as it promotes long-distance oospore dispersal. Endoornithochory is considered the main mode of dispersal of Characeae oospores [14,15,16], which play a key role in the propagation, persistence, and colonisation of (new) habitats [17].

Within the frame of a European project (Developing strategies for the protection of taxa consisting of interconnected sexual and parthenogenetic reproducing strains-ProPartS-, Biodiversa+, [4]), which aims to characterise the gene flow between *C. canescens* populations using population-genetic analyses, in combination with research into ecological niche structure and the restoration potential of former habitats, interfertility experiments between female and male specimens from different origins were conducted. Hybridisation experiments in the laboratory with macroalgae, particularly those of interest in aquaculture, are frequent ([18,19] and references therein). However, such studies are scarce for charophytes. In this arena are the seminal studies of Proctor [20,21,22] and others [23,24] (performed with different taxa within the genus *Chara*, which resulted in successful crosses between different “species”, but also the identification of reproductive isolation among populations belonging to the same “species”. Later, Blindow et al. [25] assessed the reproductive isolation between freshwater and brackish water populations of the dioecious charophyte *Chara aspera* by means of cross-fertilisation experiments and Amplified Fragment Length Polymorphism (AFLP) analyses. In this line, our study was aimed to be the first to explore interregional fertility in *C. canescens* to shed light on the population connectivity of this peculiar species. This study reports preliminary results while detailing the experimental difficulties encountered with this species of charophyte, providing essential information to optimise future experimental designs.

## 2. Results

### 2.1. Interfertility Trials with Germinated Specimens from Sediment Oospores

**Figure 1:**
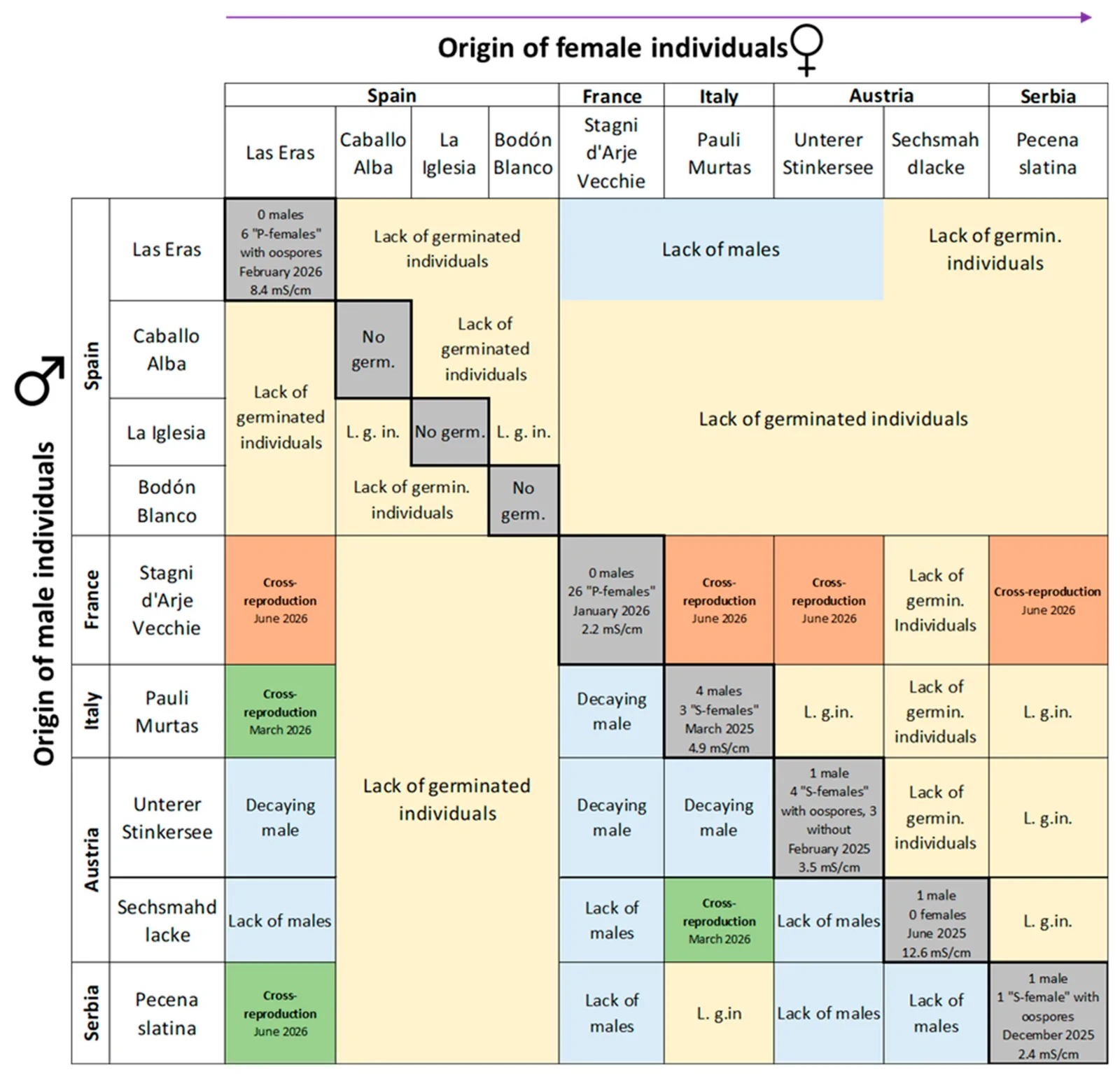
Summary of theoretical interfertility tests between European sexual populations of *Chara canescens*. The origin of individuals is ordered by the increasing geographical longitude of the sites (purple arrow). The description of the results from the germination of sediment oospores (number of germinated male individuals, type of germinated females—“P-females”: supposedly parthenogenetic females; “S-females”: supposedly sexual females), with some other details (data of the end of germination trials, mean water conductivity of the cultures) is presented diagonally, coloured in grey and outlined in black. The performed cross-reproduction trials are indicated: green colour represents interfertility trials with specimens germinated in the lab; orange indicates interfertility trials with specimens collected in the field (at least one of the sexes). The reasons that prevented the other potential interfertility tests are also indicated: light-yellow for the lack of germinated individuals (L. g. in.), and light-blue for lack of males or males in decaying status.

Out of 72 possible cross-reproduction experiments with germinated specimens from sediment oospores, only seven of them could be performed (Figure 1).

**Figure 2:**
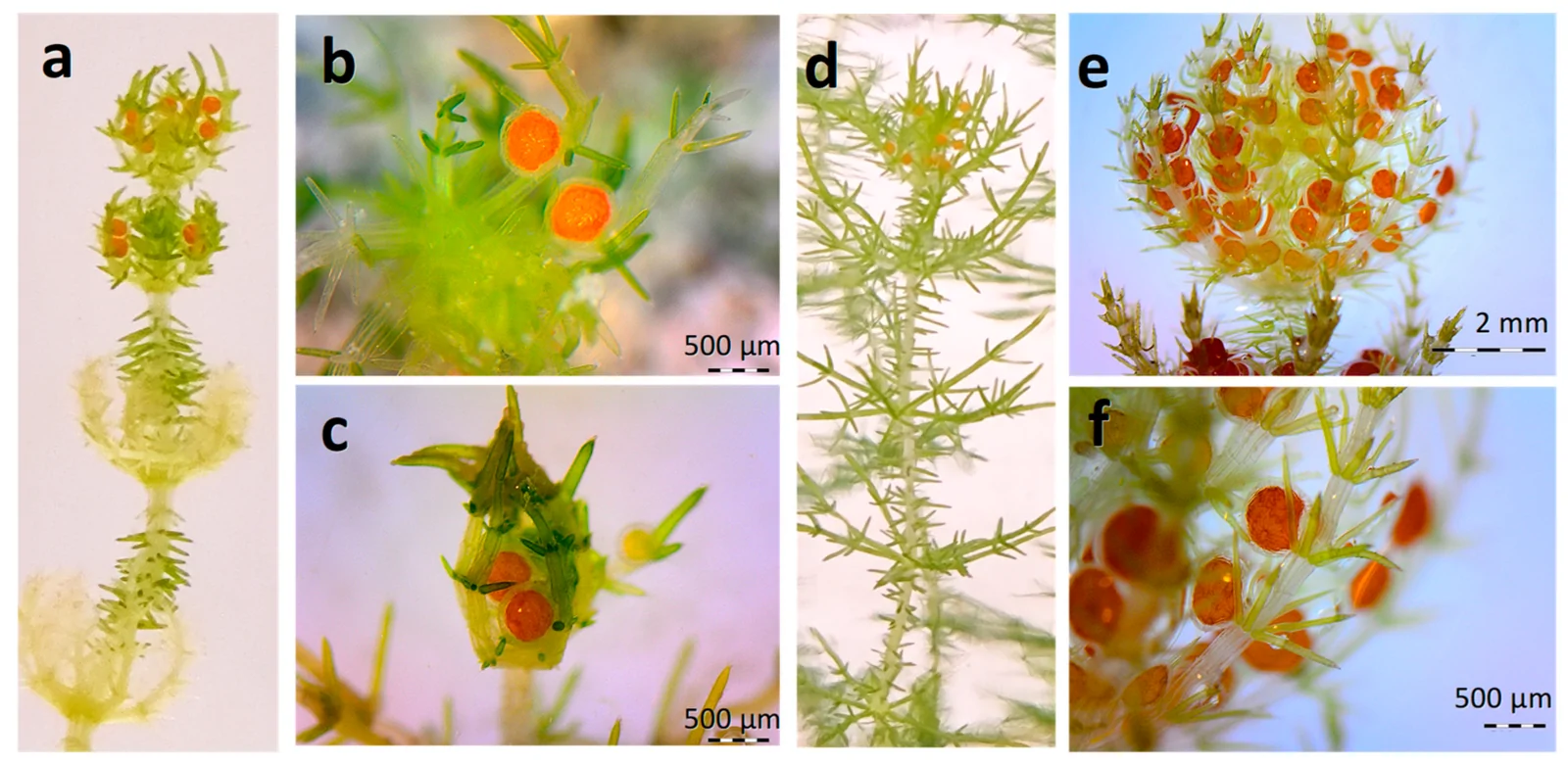
Photographs of *Chara canescens* male specimens (showing the antheridia) germinated from sediment oospores of: (**a**) Pauli Murtas (Sardinia, Italy), (**b**) Unterer Stinkersee (Austria), (**c**) Sechsmahdlacke (Austria), (**d**) Pečena Slatina (Serbia), or collected in the field, (**e**) Stagni d’Arje Vecchie (Corsica, France), (**f**) detail of the antheridia of the specimen from Stagni d’Arje Vecchie shown in (**e**).

For all sites, the number of germinated female individuals was much higher than the number of male specimens. From the sediments, a total of four male specimens germinated from the Pauli Murtas wetland (Sardinia) (Figure 1 and Figure 2a). Only one male individual from each Austrian location germinated (Figure 1 and Figure 2b,c) and also only one male from the sediments of the Serbian location (Figure 1 and Figure 2d). From the four Spanish small water bodies, the only germination was obtained from Las Eras, but no males appeared, and from the French sediment (Stagni d’Arje Vecchie, in Corsica) no males germinated either (Figure 1). With this scarce material, the following crossings could be performed (marked in green in Figure 1):

Crossing 1. One male from Sechsmahdlacke (Austria) × 20 females from Pauli Murtas (Italy):

After 4 months, eight females (40%) developed oospores. The collected F_1_ oospores subjected to germination (in April 2026) did not germinate after 4 months.

Crossing 2. One male from Pauli Murtas (Italy) × 10 females from Las Eras (Spain):

After 4 months, only three tiny female individuals (30%) with less than 20 oospores per individual appeared; the rest were branched specimens without sexual organs. No F_1_ oospores were collected, because the tiny individuals were considered to be more important for genetic analyses, and the extraction of oospores could have damaged them.

Crossing 3. One male from Pečena Slatina (Serbia) × 3 females from Las Eras (Spain):

After 2 and a half months, only one female (33%) with oospores appeared. No F_1_ oospores were collected, due to the low number of produced oospores.

### 2.2. Interfertility Trials with Collected Specimens from the Field

The cross-reproduction experiments with male (Figure 2e,f) and female specimens collected directly from Stagni d’Arje Vecchie (Corsica) and sent to Valencia, referenced in Figure 1 in orange, allowed the following combinations, and the results after 2 and a half months are:

Crossing 4. Twelve males from Stagni d’Arje Vecchie (Corsica) × 9 females from Las Eras (Spain):

Only one female appeared with oospores (11%). No F_1_ oospores were collected, due to the low number of produced oospores.

Crossing 5. Twelve males from Stagni d’Arje Vecchie (Corsica) × three females from Pauli Murtas (Italy):

No females with oogonia or oospores were detected.

Crossing 6. Ten males from Stagni d’Arje Vecchie (Corsica) × five females from Unterer Stinkersee (Austria):

Three females (60%) with oospores appeared. No F_1_ oospores were collected, due to the low number of produced oospores.

Crossing 7: Ten males from Stagni d’Arje Vecchie (Corsica) × two females from Pečena Slatina (Serbia):

No females with oogonia or oospores were detected.

## 3. Discussion

Performing interfertility laboratory experiments with *Chara canescens* specimens, both from the field and germinated from sediments, has been a challenging task (Table 1). We analyse the different causes.

### 3.1. The Use of Germinated Specimens in the Laboratory

Among the main problems we encountered while trying to conduct interfertility experiments was the low number of individuals germinated from the sediment oospores, and, particularly, the very low number of male specimens, independently of the sex ratio found in the populations living in the water column of the respective sites (for example, in the Austrian populations the sex ratio was 50:50, [10,26]). We do not consider the salinity conditions in the cultures to be a problem, since the conductivity values during the germination trials were similar to the values found in the original locations. For example, in the Las Eras water body, the conductivity range was 8–13 mS/cm [27], and 8.4 mS/cm in the cultures; in the case of specimens from Pauli Murtas, despite *C. canescens* being found living under extreme salinity conditions (63 mS/cm, when the water body was drying up) in the field [28], in laboratory conditions, the male developed at a conductivity of 5 mS/cm; the original conductivity in Pečena Slatina was 6.7 mS/cm in the field, while in laboratory conditions, the male developed at 2 mS/cm; in the laboratory, the male individual of Sechsmahdlacke grew at a conductivity value of 13 mS/cm, and currently this water body is facing huge changes in conductivity during the dry-rewetting process. The germination rate might be higher if the conductivity conditions in the laboratory were matching those of the field. However, a more important issue could be not just conductivity (salinity) but the specific ion composition. This should be considered in further research. It has been described how high (29 °C) temperatures negatively affected charophyte germination [29]. In our experiments, temperature was much lower (22 °C) and within the range found in most water bodies in the favourable season. Then, no negative effects of temperature were expected. During the germination trials, the measured water pH showed a mean value of 8.2 ± 0.3, which is within the known range for the species, although higher values have been found in the water bodies for the Spanish sexual populations [27].

Scarce information about the factors affecting *C. canescens* formation of the sexes exists in the literature. Ernst, in 1921 [30], proposed Mg^2+^ ions to influence this formation. Winter and Kirst, in 1991 [31], also suggested Mg^2+^ ions playing a role in sex allocation (while not playing a role in turgor regulation, since their intracellular level was kept constant over the whole range of salinities tested). However, the above-cited experiments were performed with parthenogenetic organisms, and hence only the “sex” allocation can be debated between investing in forming asexual oospores or vegetative growth. Schaible and Schubert, in 2008 [7], compared a parthenogenetic population with a sexual one, studying the potential responsibility of differences in salinity or irradiance acclimation abilities in the absence of male organisms in the parthenogenetic populations, but no sex-specific ecophysiological characteristics could be found.

Another procedural drawback was the long (also variable) time between when the germination trial was set up and the availability of appropriate individuals for the crossings (from one to three months). This variability for the different populations implies that even when flooding sediments in the laboratory from different origins at the same time, the germinated organisms appeared desynchronized.

The sympatric occurrence of *C. canescens* sexually and parthenogenetically reproducing females in Neusiedler See-Seewinkel (Austria) was previously described by Schaible et al. [10,32] using a molecular marker and a demographic survey. Their results suggested that sympatry had been present for more than a century. Now, we can corroborate, with another type of evidence, that, at least in the Las Eras water body (Spain), the *C. canescens* population is sexual (with both females and males; [27]), but is also formed by both types of females, because of the appearance of oospores on female specimens germinated from the sediment, in the absence of male individuals. This circumstance further complicated the possibility of being sure in reproduction crossings 2, 3 and 4 that the obtained oospores had originated from cross-fertilisation, since it was impossible to visually distinguish between parthenogenetic and sexual females when selected. The same problem occurred with crossing 6, with the females of Unterer Stinkersee (Austria), with the probable existence of sexually and parthenogenetically reproducing females [32].

### 3.2. The Use of Specimens Directly Collected from the Field

The number of sexual populations in Europe is low, but, moreover, these few European *C. canescens* sexual populations are located a great distance from each other (for example, Central Spain-Neudisedler See Seewinkle National Park: 2400 km; Pauli Murtas-Pečena Slatina: 1680 km, etc.); therefore, the simultaneous samplings in at least two sites to get living specimens of both sexes to perform the interfertility analyses is difficult. On the other hand, in other sites historically described as possessing *C. canescens* sexual populations, such as Lago di Pergusa in Sicily [33], and Lucio Largo (Doñana National Park, in Spain [34]), sexual individuals could not be found during our prospections, nor did sexual specimens germinate from flooded sediment in the laboratory. Hence, no crossing trials could be carried out with specimens from these origins. Moreover, the Mediterranean areas suffer common water deficits, as a combination of summer dryness and decadal precipitation variability [35], and many sites had dried up in recent years; thus, no living individuals could be taken during the planned samplings.

In addition, in the field, the sex ratio male–female was clearly biased toward female individuals (for example, in the Spanish water bodies it ranged from 60:40 in Las Eras, 5:95 in Bodón Blanco, 10:90 in Caballo Alba and 25:75 in La Iglesia, [27]). Hence, the availability of male specimens was low.

Although some species of charophytes can be easily cultivated by planting apical parts of the field-collected specimens in sediments under laboratory conditions (i.e., *Chara vulgaris*, *C. hispida*, etc.; see [36]), in the case of *C. canescens,* its vegetative growth is not easy to obtain (personal observation); so, obtaining fructified “healthy” cultures in the laboratory for the cross-reproduction experiments was not satisfactory. The habitat of freshwater species is easier to reproduce in laboratory conditions, while a brackish water environment requires more adjustments, not only to total salinity but also to ion-specific composition [37]. We have corroborated that collecting samples of living specimens in the field and sending them by courier/mail affects the probability of getting successful cultures for the experiments.

Considering all the above-mentioned reasons, we had to resort to using mainly germinated specimens from the sediments for the experiments.

### 3.3. Cross-Fertilisation in Chara canescens

In other charophyte species, such as *Chara aspera*, a lack of genetic isolation was found between brackish and freshwater specimens [25], and the authors explained this by the fact that both the brackish (Baltic Sea) and freshwater sites (in Sweden) have evolved rather recently, being accessible for colonisation for the first time after the last glacial period by long transport distances of waterfowl [14]. However, Croy [24] found reproductive isolation for the same species collected from different freshwater sites from regions that were ice-free during the last glacial period (Spain, France, the Netherlands and United Kingdom). Unfortunately, in the case of *C. canescens*, despite the efforts put into this topic, this information is still lacking. Out of all the possible cross-reproduction experiments (72 combinations), not only could very few be performed (seven combinations), only five of them produced oospores (71%), and only Crossing 1, with males from Sechsmahdlacke (Austria) and females from Pauli Murtas (Italy), produced supposedly F_1_ sexual oospores. Moreover, this new generation could not be genetically studied because such oospores, when subjected to germination, unfortunately did not germinate, despite having been dried to break dormancy [38,39].

## 4. Materials and Methods

**Figure 3:**
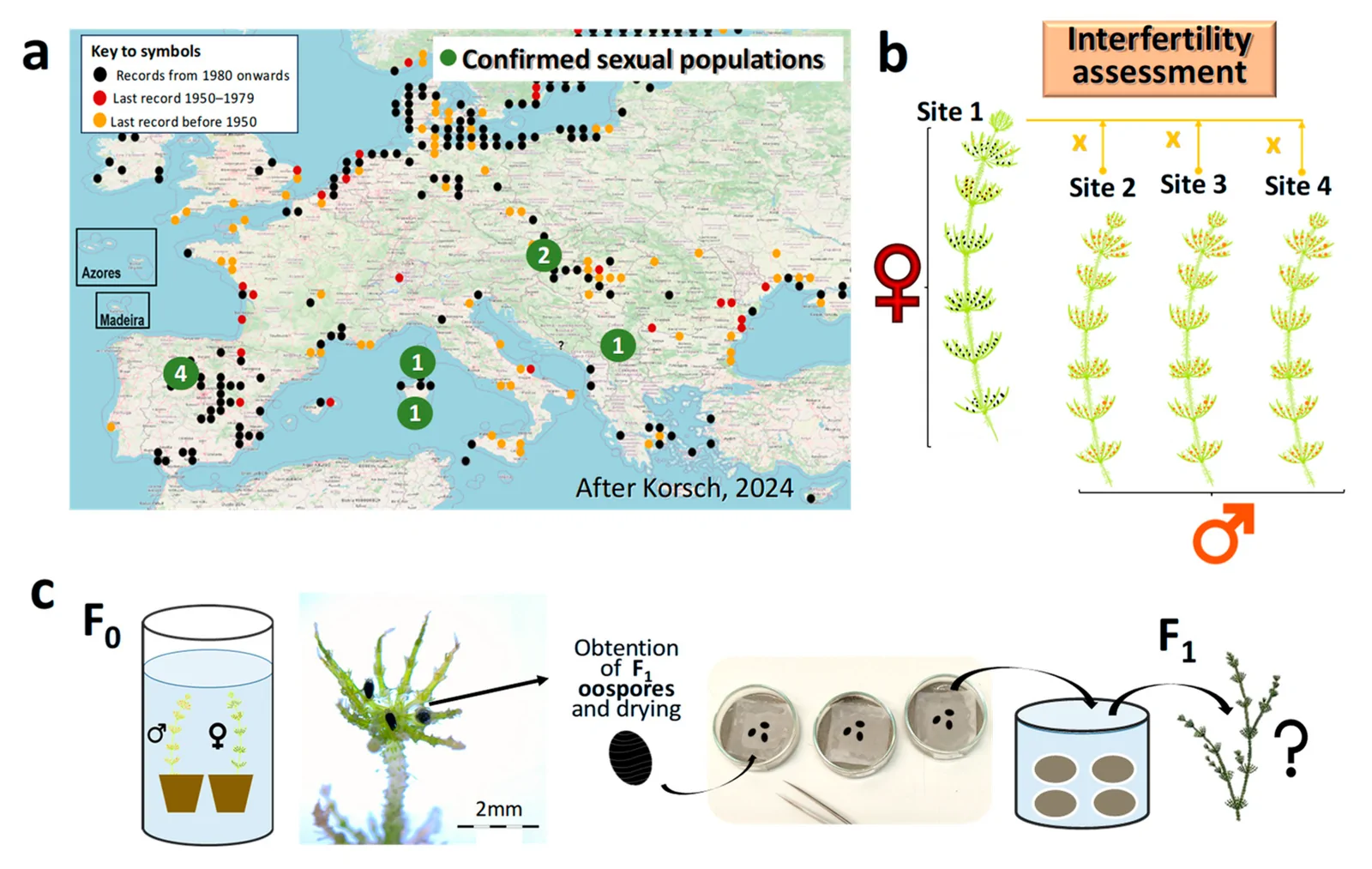
(**a**) Map of the sites with sexual populations of *Chara canescens* (green dots) from where sediment samples were taken for the interfertility experiments. The number inside green dots indicates the number of populations in each area. (**b**) Scheme of the tests to be performed: crossing males from different regions with females from other sites. (**c**) Scheme of germination test for F_1_ oospores.

Sediment samples were obtained from 9 water bodies in 5 countries which contained *C. canescens* sexual populations (see Figure 3a): Spain (the Las Eras, Caballo Alba, La Iglesia and Bodón Blanco water bodies, [27]), France (Stagni d’Arje Vecchie in Corsica, [40], E. Lambert, pers. comm.), Italy (Pauli Murtas, Sardinia, [41]), Austria (ponds of the Neusiedler See Seewinkel National Park and Sechsmahdlacke, [26]) and Serbia (Pečena Slatina, [42]). Main information about the different water bodies can be found in Table 2. All the samples were sent from the respective countries to be gathered in the Integrative Ecology lab of the Cavanilles Institute for Biodiversity and Evolutionary Biology (University of Valencia), where the interfertility experiments were carried out in the framework of the European ProPartS project. There, the sediment was allowed to dry out, spreading it on a surface, at room temperature and in darkness. The dry sediment samples were placed at the base of plastic containers and covered with water (a mixture of dechlorinated tap water and water taken at the respective sites when possible; otherwise, artificial saline water was prepared). The containers were paced on the shelves of a cultivation room (Photosynthetically active radiation: 30–50 µmol photons/m^2^ s provided by Sylvania Gro-Lux F58W tubes, manufactured by Feilo Sylvania Spain in Madrid; Light:Darkness 13:11; temperature: 22 °C). Water was added periodically to compensate for evaporation. The water conductivity of the cultures was checked periodically by means of a portable meter (HANNA Tester CE/TDS/Temperature-waterproof Ref. HI98312, Woonsocket, RI, USA) to ensure the values were within the optimum range for the study species. In case of measuring too low conductivity values, a dissolution of synthetic seawater (Instant Ocean^®^, Aquarium Systems, Sarrebourg, France) salts, mixed with dechlorinated tap water, was added to the cultures, when necessary, to increase the conductivity. Additionally, water pH values were measured occasionally. Flooded sediments were observed weekly for the appearance of germlings.

When the sex of the germinated specimens could be determined by the existence of fructifications (oogonia in the females and antheridia in the males), one or several specimens of each sex were isolated (i.e., F_0_ female, which represents the original parent generation in genetic breeding generations) (with a portion of sediment) and placed in a new container with one or several individuals of the other sex (i.e., F_0_ male) from a different origin (Figure 3b). The water in this container was a mixture of filtered water from both cultures.

When cross-fertilisation was produced, the oospores (F_1_ or first filial generation) of the female specimens were collected, allowed to dry out and then subjected to a germination experiment (inside Nytal filter packages, covered with sediment from the original culture and then flooded; [29]) (Figure 3c), to analyse the F_1_ offsprings.

Most of the F_0_ female specimens were prepared for genetic analyses after the crossings and sent to the genetic lab of the Department of Ecosystem Management, Climate and Biodiversity at the BOKU University in Vienna, where *C. canescens* population genetics is investigated to conclude about past and recent gene flow between sexual and parthenogenetic populations.

## Figures and Tables

**Table 1:** List of drawbacks found responsible for the unsuccessful interfertility trials.

With *C. canescens* Specimens Germinated in the Laboratory	With *C. canescens* Specimens Collected from the Field
Relatively low number of germinated individuals in general	Low number of sexual populations in Europe
Very low number of male individuals (sex ratio clearly biased to females)	Long distances between sexual populations to obtain specimens simultaneously for the crossings
Long time for germination from the sediment oospores and asynchrony between populations of different origin	Low number of male individuals in the population (sex ratio biased to females)
Possible sympatric existence of both types of females in the same site and impossibility to visually distinguish among them	Difficulties in getting “healthy” cultures in the laboratory

**Table 2:** Main features of the water bodies considered for the interfertility trials with *Chara canescens*. Type of habitat: inner (i) or coastal (c), temporary (t) or permanent (p). Country codes: Spain (SP), France (FR), Italy (IT), Austria (AT), Serbia (RS). Water depth, conductivity and pH indicate the values when sampling.

Country	Area (ha)	Type of Habitat	Water Depth (cm)	Conductivity (mS/cm)	pH	Reference
Las Eras (SP)	7	i/t	40	8.9	10.7	[27]
Caballo Alba (SP)	16	i/t	35	8.5	10.3	[27]
La Iglesia (SP)	8	i/t	85	5.9	10.1	[27]
Bodón Blanco (SP)	7	i/t	20	4.2	9.6	[27]
Stagni d’Arje Vecchie (FR)	4	c/t	20	3.3	-	Unpublished
Pauli Murtas (IT)	26	i/t	10	63	-	[28]
Unterer stinkersee (AT)	27	i/t	25	8.5	-	Unpublished
Sechsmahdlacke (AT)	10	i/t	30	-	-	Unpublished
Pečena Slatina (RS)	153	i/p	-	6.7	8–9	Unpublished

## Data Availability

The raw data supporting the conclusions of this article will be made available by the authors on request.

## Abbreviations

The following abbreviations are used in this manuscript:

ProPartS	Acronym of the Biodiversa+ project “Developing strategies for the protection of taxa consisting of interconnected sexual and parthenogenetic reproducing strains”
F_0_	Represents the original parent generation in genetic breeding generations
F_1_	Represents their direct offspring (the first filial generation)
PAR	Photosynthetically active radiation
